# The relation between observer-rated depressive symptoms and patient-rated quality of life after six weeks of antidepressant treatment: pooled patient-level analysis of mirtazapine trials

**DOI:** 10.1192/bjo.2026.12002

**Published:** 2026-06-09

**Authors:** Helena Werin Sjögren, Evana López, Alexander Lisinski, Mikael Landén, Johan Lundberg, Fredrik Hieronymus

**Affiliations:** Department of Pharmacology, Institute of Neuroscience and Physiology, University of Gothenburg, Sweden; Department of Clinical Neuroscience, Karolinska Institute, Stockholm, Sweden; Department of Psychiatry and Neurochemistry, Institute of Neuroscience and Physiology, University of Gothenburg, Sweden; Department of Medical Epidemiology and Biostatistics, Karolinska Institute, Stockholm, Sweden; Department of Clinical Medicine, Aarhus University, Denmark

**Keywords:** Depressive disorders, antidepressants, quality of life, meta-analysis, clinical outcomes measures

## Abstract

**Background:**

The primary effect parameter in depression trials is usually a measure of depressive symptoms, e.g. the Hamilton Depression Rating Scale (HDRS). Such measures have been criticised for not covering patient-relevant domains, such as quality of life, and hence not accurately reflecting patient-experienced efficacy.

**Aims:**

To investigate the relation between clinician-rated depressive symptoms and patient-reported quality of life measured by the Quality-of-Life Enjoyment and Satisfaction Questionnaire Short Form (Q-LES-Q-SF).

**Method:**

We included data from six acute-phase trials (*n* = 918) comparing mirtazapine to another antidepressant (amitriptyline, fluoxetine, paroxetine or venlafaxine) where both HDRS and Q-LES-Q-SF had been administered. No study included a placebo arm. Correlations between instruments (scales, subscales and items) were assessed after six weeks. Q-LES-Q-SF outcomes for participants who were in HDRS-defined remission were contrasted to those from participants with more severe depressive symptoms.

**Results:**

Q-LES-Q-SF ratings correlated strongly with HDRS-17 (*r* = −0.73, *p* < 0.0001) and HDRS-6 (*r* = −0.72, *p* < 0.0001), but somewhat weaker to HDRS-11 (*r* = −0.64, *p* < 0.0001). Depressed mood (*r* = −0.66, *p* < 0.0001) and work and activities (*r* = −0.65, *p* < 0.0001) showed the strongest item-level correlations to Q-LES-Q-SF. Participants in HDRS-remission had average Q-LES-Q-SF scores on the lower end of those reported by healthy controls, whereas patients with mild depressive symptoms (or worse) had average life-quality scores corresponding to severe impairment.

**Conclusions:**

HDRS and Q-LES-Q-SF showed considerable agreement in depressed study participants treated with antidepressants, suggesting that HDRS meaningfully reflects patient-reported improvement.

Antidepressant studies have been criticised for their over-reliance on outcome measures that focus exclusively on depressive symptoms (e.g. the Hamilton Depression Rating Scale,^1^ HDRS, or the Montgomery-Åsberg Depression Rating Scale,^2^ MADRS).^3–6^ These measures, critics argue, might be misleading as they do not cover important domains relevant to patients, including medication side-effects, and hence may fail to accurately capture broader patient-centred outcomes such as functioning, well-being and quality of life.^3–6^ However, recent patient-level analyses suggest that the likely mechanism by which classical antidepressants engender drug-placebo separation is by inducing significant symptomatic improvement in a subgroup of patients, rather than by generating modest improvements across the board.^7–9^ It has been proposed, but not yet empirically demonstrated, that such subgroups likely also experience substantial gains in subjective well-being and functioning.^10^

Relatedly, it is well-established that different classes of antidepressants vary considerably in their efficacy at the symptom-level.^11–14^ For instance, compounds that primarily inhibit the serotonin reuptake transporter, such as selective serotonin reuptake inhibitors (SSRIs), show more favourable effects on affective symptoms of depression – like depressed mood, suicidality and feelings of guilt – compared with their effects on somatic symptoms or insomnia. By contrast, mirtazapine, with potent histamine H_1_-blocking properties, has beneficial effects on insomnia that rival, or even exceed, its effects on affective symptoms of depression.^15,16^ It remains unknown if symptom-level differences in efficacy across antidepressants translate to meaningful differences in quality of life and/or functioning. For example, depressed mood – generally the symptom most responsive to SSRIs in comparison with placebo^11–14^ – has been ranked by depressed patients as both the most^17^ important, and the least^5^ important, symptom domain, depending on analysis strategy.

## Overview and aims

To investigate the relationship between clinician-rated depressive symptoms and patient-reported outcomes such as functioning, quality of life and well-being, we assembled a set of acute-phase trials (6 trials, *n* = 918) from the development programme of mirtazapine. These trials included both HDRS scores and responses to the 16-item short form of the Quality of Life Enjoyment and Satisfaction Questionnaire (Q-LES-Q-SF).^18^ The trials compared mirtazapine with either an SSRI (*n* = 4, fluoxetine or paroxetine), the tricyclic antidepressant amitriptyline (*n* = 1) or the serotonin–noradrenaline reuptake inhibitor (SNRI) venlafaxine (*n* = 1). None of these trials included a placebo control. The primary aim was to assess the extent to which HDRS-based outcomes (with particular focus on symptomatic remission) correlated to Q-LES-Q-SF scores (including individual Q-LES-Q-SF items). As secondary aims, associations between individual HDRS symptoms and Q-LES-Q-SF scores were assessed, as was the extent to which symptom-level differences in depressive symptoms previously demonstrated for SSRIs and mirtazapine^16^ translated to Q-LES-Q-SF ratings.

## Method

### Data acquisition

In a previous patient-level meta-analysis, we assessed the symptom-level effects of mirtazapine compared with placebo and several other antidepressant classes.^16^ The data for this study consist of those studies included in the previous analysis which had utilised the Q-LES-Q-SF. As for the previous study, data were requested from the Merck Sharp & Dohme (MSD) (Rahway, NY, USA) EngageZone portal and later moved to Vivli.org. Full details on the data acquisition process have been published elsewhere.^16^

### Missing data

Fifteen subjects with HDRS ratings had no Q-LES-Q-SF reports at any time point. These subjects were excluded from all analyses. For the remaining 903 subjects, there was almost no missing data for HDRS items (<0.5%) and very little missing data (<2% in total, frequently 0%) for most Q-LES-Q-SF items. One exception was Q-LES-Q-SF item 15, which addresses ‘medication satisfaction’. Item 15 had over 50% missing data at baseline in some studies. This item may, however, be left blank at baseline, and had likely often been done so intentionally in some studies. Consequently, missing data were handled by median imputation for all HDRS and Q-LES-Q-SF items, except Q-LES-Q-SF item 15 which was instead analysed without adjusting for baseline score.

### Analyses and statistics

All analyses were carried out on the intention-to-treat, last observation carried forward (LOCF) population. Since all studies had a week six evaluation – and as this should be sufficient to demonstrate antidepressant efficacy – week six was selected as the end-point. For analyses relating Q-LES-Q-SF scores to HDRS scores, all cases were pooled regardless of which antidepressant (mirtazapine, fluoxetine, paroxetine, venlafaxine or amitriptyline) had been administered.

To enable comparisons with reported community norms,^18^ scores for the first 14 items on the Q-LES-Q-SF (1: Physical health, 2: Mood, 3: Work, 4: Household activities, 5: Social relationships, 6: Family relationships, 7: Leisure activities, 8: Daily functioning, 9: Sexual drive, interest and/or performance, 10: Finances, 11: Housing, 12: Mobility, 13: Vision, 14: Well-being) were summed together, 14 points were subtracted from the total and the result was multiplied by 100/56 (i.e. the scores were rescaled to 0 to 100% of the maximum possible score). Notably, there is some confusion in the literature with some papers having erroneously rescaled Q-LES-Q-SF scores to fall between 20 and 100% (by dividing the raw score by 70).^19,20^ The cut-offs used in these papers are based on the report by IsHak and co-workers,^21^ where scores higher than one s.d. below the community average (67) indicate normality, and scores lower than two s.d. below the community average (55.7) indicate severe impairment.

Certain items in the Q-LES-Q-SF are less likely to be responsive to short-term antidepressant treatment, either because they lack a strong causal link to the depressive episode (e.g. items 1: Physical health, 12: Mobility and 13: Vision) and/or because they represent outcomes that typically require more than six weeks to show substantial change (e.g. items 10: Finances and 11: Housing). Conversely, some items overlap with those measured directly by the HDRS (e.g. item 2: Mood and item 9: Sexual drive). For these reasons, we constructed an abbreviated Q-LES-Q-SF scale – here called Q-LES-Q-8 – including only those items that we *a priori* assumed to be responsive to short-term antidepressant treatment *and* were not directly assessed by the HDRS (the latter to avoid introducing artefactual correlations). The Q-LES-Q-8 consisted of items 3 to 8 and 14, as well as Q-LES-Q-SF item 16: Overall life satisfaction. While our item selection was based on *a priori* assumptions and results should be seen as exploratory, it closely mirrored the Mini-Q-LES-Q, which we were unaware of when designing our subscale, the only difference being that the Q-LES-Q-8 also includes item 16.^22^

We first report normalised Q-LES-Q-SF scores stratified by the HDRS-17 severity categorisation of depressed patients proposed by the American Psychiatric Association in 2000^23^ (0–7: no depression; 8–14: mild depression; 15–18: moderate depression; 19–22 severe depression; ≥23 very severe depression) and relate these to community norms. We similarly report item-level results stratified by HDRS severity category for all Q-LES-Q-SF items.

We then assessed how individual HDRS items correlated to Q-LES-Q-SF scores. These analyses were done using both the normed Q-LES-Q-SF scores and the abridged 8-item version described above. We also correlated the full HDRS-17, as well as the unidimensional HDRS-6^24^ subscale (consisting of HDRS items 1: Depressed mood, 2: Feelings of guilt, 7: Work and activities, 8: Psychomotor retardation, 10: Psychic anxiety and 13: General somatic symptoms) and its complement HDRS-11 (consisting of the HDRS-17 items not included in the HDRS-6), to the two Q-LES-Q versions.

Finally, we compared treatment outcomes between mirtazapine and a combined SSRI/SNRI group (five trials), as well as between mirtazapine and amitriptyline (one trial). For these analyses, HDRS-17-sum, HDRS-6-sum, HDRS-11-sum, Q-LES-Q-SF and Q-LES-Q-8 served as outcome parameters. These models used an analysis of covariance specification with fixed factors for trial and treatment, and the baseline score on the outcome measure included as a covariate.

All analyses were conducted remotely on the Vivli Research Environment running R version 4.4.0 for Windows (R Foundation for Statistical Computing, Vienna, Austria; https://www.r-project.org/) using only base R functions. The r-package tidyverse (version 2.0.0) was used for data management and ggplot2 (version 3.5.1) was used for producing the graphs.

### Ethics

Secondary analysis of de-identified personal data does not require ethical approval in Sweden.

## Results

Baseline characteristics for the included studies are provided in Table 1. At LOCF end-point, 228 subjects were non-depressed/in remission (≤7 HDRS points), 308 had mild depression (8 to 13 HDRS points), 179 had moderate depression (14 to 18 HDRS points), 84 had severe depression (19 to 22 HDRS points) and 104 had very severe depression (≥23 HDRS points).

**Table 1:** Included studies

Protocol	Treatment (*n*)	Age	% female	HDRS-17	HDRS-6	Depressed mood	Q-LES-Q-14	Q-LES-Q-8	Trial length (weeks)
1900011	Mirtazapine 15**–**45 mg (65) Paroxetine 20**–**40 mg (69)	44.4 (12.6)	69	25.1 (5.0)	12.1 (2.7)	2.8 (0.8)	45.6 (11.3)	26.0 (7.3)	6
1900019	Mirtazapine 15**–**60 mg (61) Fluoxetine 20**–**40 mg (65)	47.2 (15.0)	56	26.3 (4.5)	12.7 (2.5)	2.9 (0.6)	41.2 (11.8)	23.1 (7.9)	6
1900022	Mirtazapine 15**–**45 mg (12) Paroxetine 20**–**40 mg (13)	42.9 (11.9)	52	23.5 (3.6)	11.6 (1.9)	2.5 (0.5)	38.2 (10.8)	20.7 (7.1)	6
1900029	Mirtazapine 15**–**60 mg (145) Fluoxetine 20**–**40 mg (147)	43.7 (11.6)	72	28.6 (3.0)	14.0 (1.7)	3.3 (0.7)	41.5 (10.6)	23.8 (6.7)	8
1900041	Mirtazapine 15**–**60 mg (92) Amitriptyline 50**–**200 mg (98)	48.6 (9.7)	65	24.6 (3.9)	11.8 (2.3)	2.7 (0.7)	41.1 (9.3)	23.4 (5.9)	6
1900042	Mirtazapine 15**–**60 mg (77) Venlafaxine 75**–**375 mg (74)	44.9 (10.5)	66	29.2 (2.9)	14.6 (1.8)	3.2 (0.6)	38.7 (12.3)	21.4 (7.8)	8

HDRS, Hamilton Depression Rating Scale; Q-LES-Q, Quality-of-Life Enjoyment and Satisfaction Questionnaire Short Form.

### Descriptive analyses

**Fig. 1:**
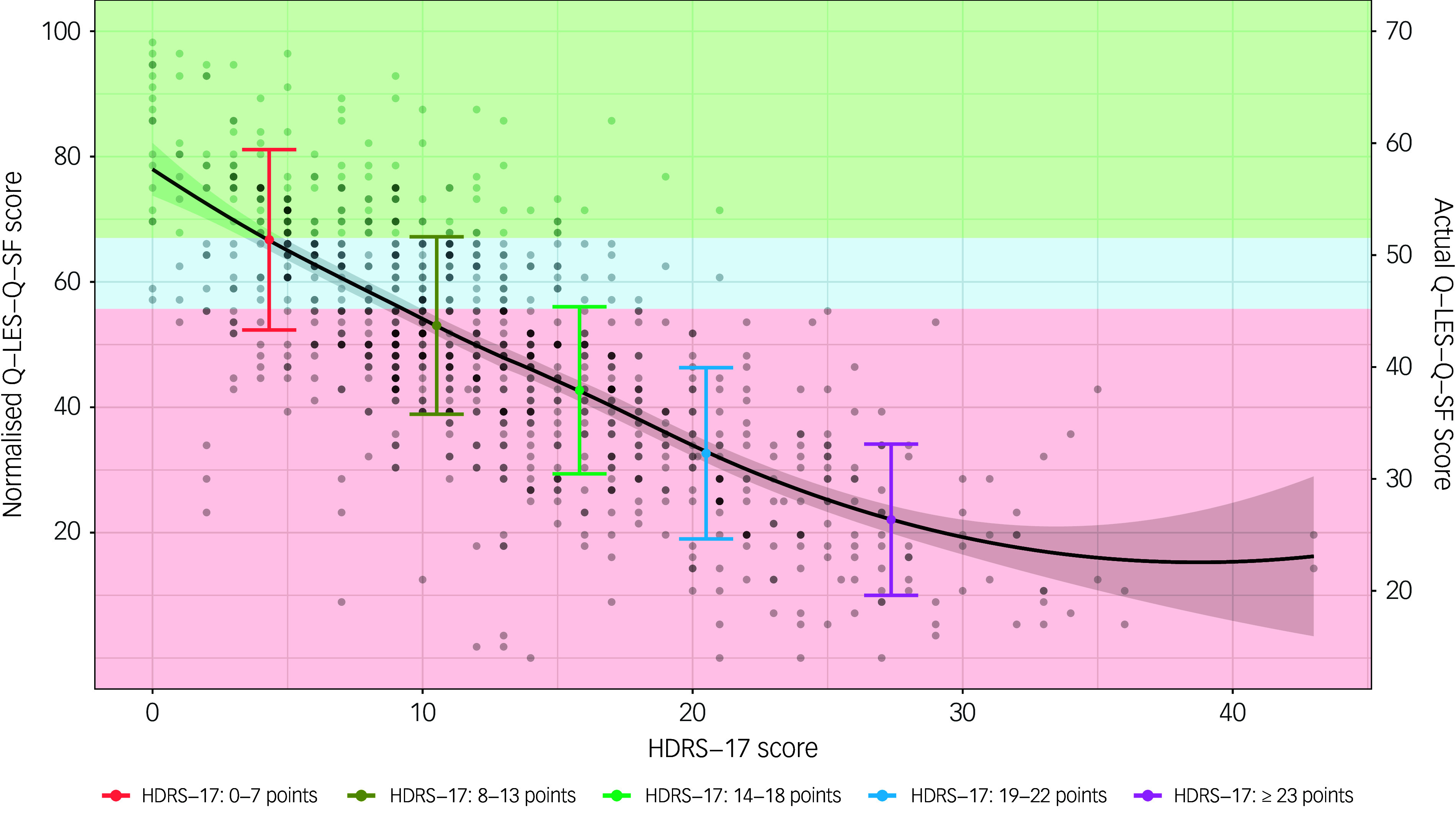
Q-LES-Q scores mapped to depression severity categories at end-point. The red field corresponds to Q-LES-Q scores −2 s.d. or lower than the community average, the blue field corresponds to −1 to −2 s.d., and the green field corresponds to scores above −1 s.d. Individual patients are shown on the scatterplot, with intensity corresponding to the number of observations. Means ± 1 s.d. Q-LES-Q-SF scores for the five HDRS subgroups are overlayed on a LOESS regression of Q-LES-Q-SF on HDRS-17. Q-LES-Q, Quality-of Life Enjoyment and Satisfaction Questionnaire; Q-LES-Q-SF, Quality-of-Life Enjoyment and Satisfaction Questionnaire Short Form; HDRS, Hamilton Depression Rating Scale; LOESS, locally estimated scatterplot smoothing.

Figure 1 shows normalised (0 to 100) and actual Q-LES-Q scores (14 to 70) plotted over end-point HDRS-17 scores for the LOCF-population. Q-LES-Q scores for the different HDRS-17 categories all separated at *p* < 0.0001. Patients in remission according to their HDRS-17 scores scored, on average (mean 66.7, median 67.9), just at the cut-off for normality (≥67). Higher HDRS scores were associated with lower life-quality scores: Patients with mild depression (HDRS end-point score: 8 to 13) scored on average (mean 53.0, median 51.8) below the cut-off for severe impairment (≤55.7), and those with very severe depressive symptoms (HDRS end-point score ≥23) scored lower yet (mean 22.1, median 19.6). While there was considerable heterogeneity, the association between remission and impaired life quality was strong. While 21% of patients in HDRS-based remission scored below the community norm for severe impairment, the corresponding proportions were 61% for mild depression, 85% for moderate depression, 95% for severe depression and 100% for very severe depression.

**Fig. 2:**
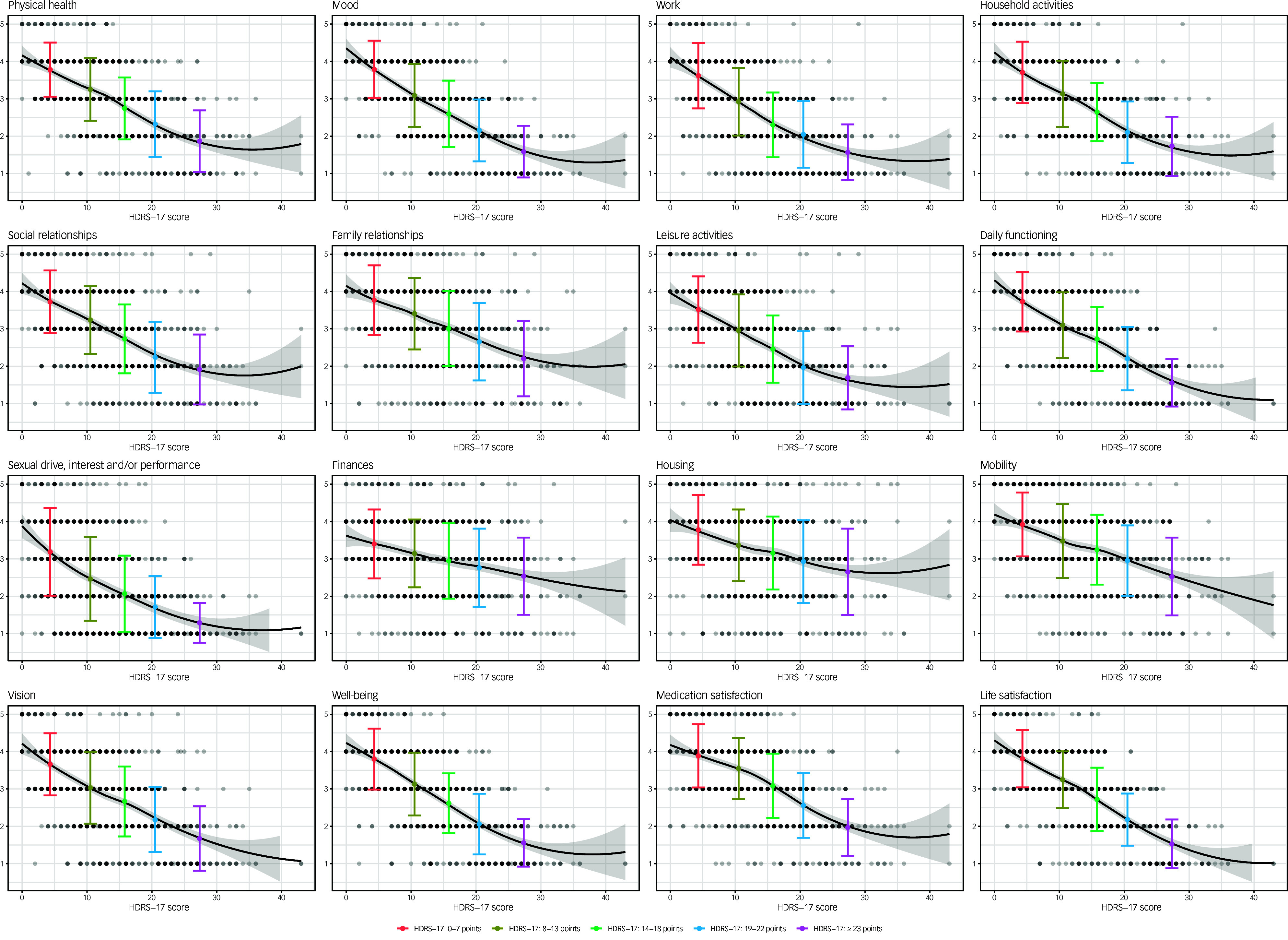
Relation between individual Q-LES-Q items and HDRS-17 symptom severity at end-point. Individual patients are shown on the scatterplot, with intensity corresponding to the number of observations. Means ± 1 s.d. Q-LES-Q item scores for the five HDRS subgroups are overlayed on a LOESS regression of Q-LES-Q item score over the HDRS-17 score. Q-LES-Q, Quality-of-Life Enjoyment and Satisfaction Questionnaire; HDRS, Hamilton Depression Rating Scale; LOESS, locally estimated scatterplot smoothing.

Figure 2 details the relation between HDRS-17 severity categories and individual Q-LES-Q items. The distribution of responses was largely the same for all items, with a clear pattern such that lower HDRS-rated severity associated with higher ratings for all Q-LES-Q items. Q-LES-Q items geared towards somatic health (‘Physical health’, ‘Mobility’ and ‘Vision’) did not show weaker associations to HDRS scores. However, while ‘Housing’ and ‘Finances’ were also inversely correlated to HDRS scores, the associations were noticeably weaker.

### Correlation analyses

Q-LES-Q-SF ratings correlated strongly with HDRS-17 (*r* = −0.73, 95% CI −0.70 to −0.76; *p* < 0.0001; single-trial range −0.84 to −0.64), with HDRS-6 (*r* = −0.72, −0.69 to −0.75; *p* < 0.0001; −0.81 to −0.62), but somewhat weaker with HDRS-11 (*r* = −0.64, −0.60 to −0.67; *p* < 0.0001; −0.73 to −0.58). Substituting the Q-LES-Q-SF for the shorter Q-LES-Q-8 did not materially affect the associations (HDRS-17 *r*: −0.73, HDRS-6 *r*: −0.73, HDRS-11 *r*: −0.62; all *p* < 0.0001).

Table 2 details correlations between individual HDRS-17 items and Q-LES-Q scores. While all HDRS items correlated significantly to Q-LES-Q scores, those belonging to the HDRS-6 cluster were generally found to have the strongest correlations. Four of the HDRS-6 items, ‘Depressed mood’ (*r* = −0.66), ‘Work and activities’ (*r* = −0.65), ‘Psychic anxiety’ (*r* = −0.49) and ‘Guilt’ (*r* = −0.48) showed the strongest correlations to Q-LES-Q-SF scores and the remaining two HDRS-6 items were found at position 7 (‘Psychomotor retardation’, *r* = −0.42) and 9 (‘General somatic symptoms’, *r* = −0.40). ‘Somatic anxiety’ (rank 5, *r* = −0.46), ‘Suicide’ (rank 6, *r* = −0.44) and ‘Gastrointestinal symptoms’ (rank 8, *r* = −0.41) were those belonging to the HDRS-11 cluster that placed better than the two HDRS-6 items that were not among the four highest ranked items. Only two items were found to have a correlation below *r* = −0.28: ‘Insight’ at *r* = −0.10 and ‘Weight loss’ at *r* = −0.07. Replacing the Q-LES-Q-SF with the Q-LES-Q-8 did not materially affect the results.

**Table 2:** Correlations between HDRS-17 items and Q-LES-Q scores at end-point

Item	Q-LES-Q-14 *r* (95% CI); *p:* single-trial range	Q-LES-Q-8 *r* (95% CI); *p*: single-trial range
Depressed mood	−0.66 (−0.62 to −0.69); *p* < 0.0001; −0.75 to −0.53	−0.67 (−0.63 to −0.70); *p* < 0.0001; −0.74 to −0.55
Guilt	−0.48 (−0.43 to −0.53); *p* < 0.0001; −0.58 to −0.38	−0.50 (−0.45 to −0.55); *p* < 0.0001; −0.61 to −0.39
Suicide	−0.44 (−0.39 to −0.49); *p* < 0.0001; −0.72 to −0.34	−0.46 (−0.40 to −0.51); *p* < 0.0001; −0.73 to −0.36
Initial insomnia	−0.34 (−0.28 to −0.39); *p* < 0.0001; −0.39 to −0.26	−0.33 (−0.27 to −0.39); *p* < 0.0001; −0.41 to −0.26
Middle insomnia	−0.33 (−0.27 to −0.38); *p* < 0.0001; −0.50 to −0.11	−0.34 (−0.28 to −0.40); *p* < 0.0001; −0.53 to −0.08
Late insomnia	−0.34 (−0.28 to −0.39); *p* < 0.0001; −0.39 to −0.20	−0.34 (−0.28 to −0.49); *p* < 0.0001; −0.38 to −0.19
Work and activities	−0.65 (−0.61 to −0.69); *p* < 0.0001; −0.74 to −0.58	−0.66 (−0.62 to −0.70); *p* < 0.0001; −0.75 to −0.58
Psychomotor retardation	−0.42 (−0.36 to −0.47); *p* < 0.0001; −0.57 to −0.23	−0.41 (−0.36 to −0.47); *p* < 0.0001; −0.55 to −0.21
Psychomotor agitation	−0.28 (−0.22 to −0.34); *p* < 0.0001; −0.39 to −0.19	−0.27 (−0.20 to −0.33); *p* < 0.0001; −0.39 to −0.17
Psychic anxiety	−0.49 (−0.43 to −0.53); *p* < 0.0001; −0.61 to −0.41	−0.49 (−0.44 to −0.54); *p* < 0.0001; −0.62 to −0.42
Somatic anxiety	−0.46 (−0.40 to −0.51); *p* < 0.0001; −0.54 to −0.34	−0.45 (−0.40 to −0.50); *p* < 0.0001; −0.51 to −0.33
Gastrointestinal symptoms	−0.41 (−0.35 to −0.46); *p* < 0.0001; −0.49 to −0.24	−0.40 (−0.34 to −0.45); *p* < 0.0001; −0.48 to −0.24
General somatic symptoms	−0.40 (−0.34 to −0.45); *p* < 0.0001; −0.70 to −0.31	−0.41 (−0.35 to −0.46); *p* < 0.0001; −0.76 to −0.33
Genital symptoms	−0.37 (−0.31 to −0.43); *p* < 0.0001; −0.59 to −0.24	−0.35 (−0.29 to −0.40); *p* < 0.0001; −0.61 to −0.23
Hypochondriasis	−0.35 (−0.29 to −0.41); *p* < 0.0001; −0.57 to −0.25	−0.33 (−0.27 to −0.39); *p* < 0.0001; −0.50 to −0.21
Weight loss	−0.07 (−0.01 to −0.13); *p* = 0.0269; −0.37 to 0.03	−0.07 (−0.002 to −0.13); *p* = 0.0441; −0.35 to 0.06
Insight	−0.10 (−0.04 to −0.16); *p* = 0.0025; −0.37 to 0.01	−0.10 (−0.03 to −0.16); *p* = 0.0034; −0.37 to 0.01

HDRS, Hamilton Depression Rating Scale; Q-LES-Q, Quality-of-Life Enjoyment and Satisfaction Questionnaire Short Form. Single-trial range represent the lowest and highest *r* coefficient from the six included trials when analysed separately.

### Between-treatment comparisons

Mirtazapine outperformed the combined SSRI/SNRI group (5 trials) on HDRS-17 (mean difference: 1.17 points, *p* = 0.0442) and on HDRS-11 (mean difference: 1.081 points, *p* = 0.0005), but not on HDRS-6 (mean difference: 0.102 points, *p* = 0.840). The observed superiority on HDRS-17 and HDRS-11 did not translate into higher quality of life as measured by Q-LES-Q-SF (mean difference: −0.112 points, *p* = 0.891) or Q-LES-Q-8 (mean difference: −0.048 points, *p* = 0.929).

Mirtazapine did not differ from amitriptyline on HDRS-17 (mean difference: −0.657 points; *p* = 0.467), HDRS-6 (mean difference: −0.368 points; *p* = 0.441), HDRS-11 (mean difference: −0.326 points, *p* = 0.507), or Q-LES-Q-14 (mean difference: 1.98 points, *p* = 0.110). Q-LES-Q-8 scores were higher for amitriptyline (mean difference: 1.604 points; *p* = 0.045).

## Discussion

The antidepressant literature has been criticised for focusing too narrowly on depressive symptoms and, by so doing, potentially providing a biased view of antidepressant treatment effects which is not aligned with patient-experienced benefits and risks.^3–6^ The primary finding of the present work, i.e. that HDRS-17 ratings correlate strongly (*r* = 0.73) to patient-reported quality of life as rated by the Q-LES-Q-SF, should partly alleviate such concerns. Specifically, while patients who were in HDRS-defined remission reported Q-LES-Q-SF scores that were at the lower end of what has been reported for healthy controls, study participants rated as ‘mildly depressed’ instead showed scores that were, on average, compatible with severe impairment (Fig. 1). The strong correlations between measures also extended to individual HDRS-17 symptoms, most notably ‘Depressed mood’ and ‘Work and activities’ (Table 2), as well as to HDRS-17 subscales. All in all, the results suggest that the HDRS-17 is likely to be a sufficiently good proxy for patient-experienced quality of life.

While agreement between measures was substantial, there was still considerable heterogeneity such that roughly one out of five patients in HDRS-defined remission considered themselves severely impaired by Q-LES-Q-SF standards. Conversely, about one in six out of those with mild HDRS-17 rated depression (HDRS end-point score 8 to 13) had scores in the Q-LES-Q-SF normal range. These data are in line with results from previous studies reporting disagreement between the HDRS-17 cut-off for remission and patient-rated remission,^25,26^ and, more generally, support the distinction between symptomatic remission and functional recovery.^27^

The HDRS-6 has previously shown larger separation between SSRIs and placebo than the full HDRS-17,^28^ but the extent to which it correlates to patient-rated quality of life has not been previously assessed. In this sample, HDRS-6 (*r* = −0.72) showed essentially the same correlation to Q-LES-Q-SF as did the HDRS-17, with the HDRS-11 correlation (*r* = −0.64) being somewhat weaker.^18^

In line with a previous analysis from our group^16^ – of which the included trials form a subset – mirtazapine outperformed a combined SSRI/SNRI group on HDRS-17-sum and HDRS-11-sum, but not on HDRS-6-sum. As in our previous analysis,^16^ this was explained by mirtazapine’s beneficial impact on sleep and appetite (the mean difference for HDRS-17-sum excluding HDRS items reflecting sleep (items 4 to 6, appetite (item 12) and weight loss (item 16) being 0.025; *p* = 0.36). That mirtazapine, despite this superiority, did not show significantly better results than the SSRI/SNRI group on quality-of-life measures is compatible with the hypothesis that the core depression symptoms captured by HDRS-6 are more relevant to patients than symptoms like insomnia and weight loss. In line with this, items belonging to the unidimensional HDRS-6 subscale in general correlated more strongly to Q-LES-Q-SF scores (Table 2).

In the one trial that compared mirtazapine with amitriptyline (combined *n* = 190), amitriptyline showed significant superiority over mirtazapine on the Q-LES-Q-8, but no significant differences on any HDRS-17-derived measures or on the Q-LES-Q-SF. While this should be interpreted cautiously due to the small sample size and it only being observed for one of the Q-LES-Q versions, it is notable both since amitriptyline is more side-effect prone than mirtazapine^29^ and since previous studies have suggested amitriptyline may be an especially potent antidepressant.^16,30,31^

In line with the analysis by Fried and co-workers, ‘Depressed mood’ and ‘Work and activities’ were the items found to be most strongly associated with quality of life (Table 2). This contrasts with patient preference studies, such as that by Zimmermann and colleagues,^5^ where depressed mood was instead ranked as the least important (according to patients) out of eight studied domains. In that study, loss of energy/fatigue was ranked as the most important domain, with over twice the magnitude compared with depressed mood (relative importance 18.5 *v.* 8.5%).

Part of the reason for this discrepancy likely stems from the way symptom domains are concretised in patient preference studies. For example, the worst possible state of depressed mood as conceptualised by Zimmerman and colleagues was ‘Feels extremely low and distressed’ whereas the corresponding worst state for ‘Loss of energy/fatigue’ was ‘Cannot start and cope with *any activities* on his/her own (e.g. getting out of bed or washing oneself)’. Had a less severe anchor for ‘Loss of energy/fatigue’, or a more severe one for depressed mood, been used, then patient preferences would likely have shifted.

### Limitations

This study has several limitations. First, the lack of placebo-arms in the included studies preclude a direct assessment of the impact of antidepressant treatment on self-reported quality of life, functioning and well-being (as rated by the Q-LES-Q-SF). While the demonstration of strong agreement between HDRS-17 and Q-LES-Q-SF indicates that the clinician-rated HDRS-17 captures features which are relevant to patients, this is merely indirect evidence. It could be the case that patients who achieve HDRS-defined remission while on placebo report more improvement on the Q-LES-Q-SF than patients who achieve HDRS-remission on antidepressants. Second, while the included material represents all studies from the development programme for mirtazapine where the HDRS and the Q-LES-Q-SF were administered in tandem, the sample size is still comparatively small. This is especially important for between-treatment comparisons (e.g. mirtazapine versus amitriptyline) which are based on subsets of the material. Third, the material only includes HDRS and Q-LES-Q-SF data. Whether the strong agreement observed here extends to other life-quality measures (e.g. World Health Organization – Quality Of Life – Brief) or depression rating scales (e.g. MADRS) remains to be investigated. In the same vein, all included studies were industry-sponsored acute-phase trials and patient characteristics at baseline were similar between trials. Pharmacological treatment trials in depression often enforce strict inclusion and exclusion criteria which limits generalisability to the broader clinical population.^32^ Whether the results would replicate in other trial settings, or in clinical practice, thus remains to be determined. Fourth, while overall agreement between measures was strong, one in five participants in HDRS-rated remission considered themselves severely impaired by Q-LES-Q-SF standards. This suggests that there are important aspects that the HDRS fails to capture (at least at the sum-score level) and highlights the need for thorough outcome assessments in depression. In summary, the results of this exploratory analysis should be considered tentative until replicated in external samples.

In conclusion, HDRS-17 scores correlate strongly to Q-LES-Q-SF scores, patients who score low on the HDRS-17 tend to have Q-LES-Q-SF scores that fall within the normal range of community samples, and patients with mild or worse depression tend to have scores compatible with severe impairment. The unidimensional HDRS-6 subscale displayed essentially the same correlation to Q-LES-Q-SF as did HDRS-17, and the HDRS items ‘Depressed mood’ and ‘Work and activities’ also correlated strongly with Q-LES-Q-SF. Taken together, the results support the validity of the HDRS-17 score, and equally so the HDRS-6 score, as a reflection of patient-relevant improvement.

## Data Availability

The individual patient data used for this study can be requested via Vivli.org.
